# Extraordinary Aggressive Behavior from the Giant Coral Reef Fish, *Bolbometopon muricatum*, in a Remote Marine Reserve

**DOI:** 10.1371/journal.pone.0038120

**Published:** 2012-06-06

**Authors:** Roldan C. Muñoz, Brian J. Zgliczynski, Joseph L. Laughlin, Bradford Z. Teer

**Affiliations:** 1 National Marine Fisheries Service, Beaufort Laboratory, National Oceanic and Atmospheric Administration, Beaufort, North Carolina, United States of America; 2 Center for Marine Biodiversity and Conservation, Scripps Institution of Oceanography, La Jolla, California, United States of America; 3 Mariculture Hawaii LLC, Ashland, Oregon, United States of America; Biodiversity Insitute of Ontario - University of Guelph, Canada

## Abstract

Human impacts to terrestrial and marine communities are widespread and typically begin with the local extirpation of large-bodied animals. In the marine environment, few pristine areas relatively free of human impact remain to provide baselines of ecosystem function and goals for restoration efforts. Recent comparisons of remote and/or protected coral reefs versus impacted sites suggest remote systems are dominated by apex predators, yet in these systems the ecological role of non-predatory, large-bodied, highly vulnerable species such as the giant bumphead parrotfish (*Bolbometopon muricatum*) has received less attention. Overfishing of *Bolbometopon* has lead to precipitous declines in population density and avoidance of humans throughout its range, contributing to its status as a candidate species under the U. S. Endangered Species Act and limiting opportunities to study unexploited populations. Here we show that extraordinary ecological processes, such as violent headbutting contests by the world’s largest parrotfish, can be revealed by studying unexploited ecosystems, such as the coral reefs of Wake Atoll where we studied an abundant population of *Bolbometopon*. *Bolbometopon* is among the largest of coral reef fishes and is a well known, charismatic species, yet to our knowledge, no scientific documentation of ritualized headbutting exists for marine fishes. Our observations of aggressive headbutting by *Bolbometopon* underscore that remote locations and marine reserves, by inhibiting negative responses to human observers and by allowing the persistence of historical conditions, can provide valuable opportunities to study ecosystems in their natural state, thereby facilitating the discovery, conservation, and interpretation of a range of sometimes remarkable behavioral and ecological processes.

## Introduction

For large-bodied species that are generally first to be extirpated following human contact [1], [2], no-take protected areas and remote locations relatively free of human impact can harbor extraordinary ecological processes. In the marine environment, few pristine areas unaffected by human activities such as fisheries exploitation remain to provide baselines of ecosystem function and goals for restoration efforts [3]. Recent comparisons of remote and/or protected coral reefs versus impacted sites suggest remote systems are dominated by apex predators [4], yet the ecological role in these systems of non-predatory, large-bodied, highly vulnerable species such as the giant bumphead parrotfish (*Bolbometopon muricatum*) has received less attention.

*Bolbometopon* is the largest herbivorous fish on coral reefs, reaching 150 cm total length (TL) and over 75 kg total weight [5]. It is slow-growing and long-lived with delayed reproduction and low replenishment rates [6], [7], [8]. As a result, even moderate levels of exploitation have led to severe declines in size-structure and abundance of populations throughout much of its range [9], [10], [11]. In addition, *Bolbometopon* often sleeps and feeds in large groups in shallow water and shows strong site fidelity, making it highly vulnerable to exploitation by night spearfishing and netting of daytime feeding schools [12], [13]. For example, night spearfishing increased with the advent of underwater flashlights in the 1970’s, and in the western Solomon Islands led to overexploitation and the disappearance of sleeping aggregations that had persisted and supported subsistence fishing for generations [14]. Overfishing has led to a general avoidance of humans; it is known as the wariest of parrotfishes and in most locations individuals are difficult to approach underwater [15]. *Bolbometopon* was listed as Vulnerable in 2007 by the International Union for Conservation of Nature (IUCN), and became a candidate species under the U. S. Endangered Species Act in 2010.

**Figure 1:**
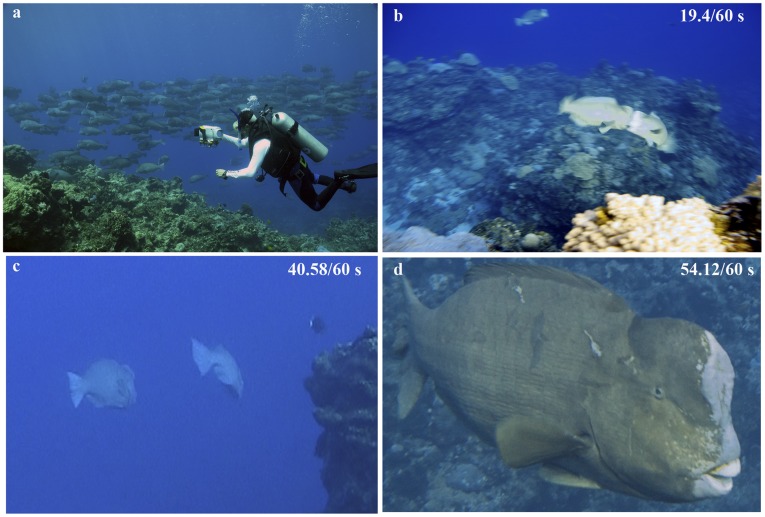
*Bolbometopon muricatum* at Wake Atoll. (**a**) Partial spawning aggregation of *Bolbometopon* consisting of 246 individuals. (**b**) Second headbutting impact. Time corresponds with video. (**c**) Capitulation by subordinate male (on right) rapidly fleeing the area with use of caudal fin following fourth charge. (**d**) Dominant male showing scale damage on back and side following headbutting bout.

One location where the ecological role of *Bolbometopon* has been studied is Australia’s Great Barrier Reef (GBR). The GBR has no commercial fisheries for parrotfishes. As such, these reefs support healthy populations of giant bumphead parrotfish where schools of 30–50 individuals can be observed regularly [6], [16]. On the GBR, individuals appear capable of bioeroding over 5 tons of reef carbonate each year [16]. Because of its large size, feeding rates, and schooling behavior, *Bolbometopon* may play a keystone role as a major coral consumer and bioeroder on coral reefs. In overfished locations, negative effects may include significant disruption to coral community structure, reductions in reef structural stability via invasive erosion by echinoids, and dramatic reductions in sediment transport [16]. Given *Bolbometopon’s* vulnerability to overexploitation and ecological role, comparative studies of its biology and ecology from additional unexploited populations are urgently needed and may provide critical insights for the development of recovery and management plans throughout its range. We studied spawning site characteristics and reproductive behavior of such a population at Wake Atoll, a U. S. Marine National Monument where great abundances of *Bolbometopon* can be commonly observed (Fig. 1a). On its spawning grounds, we witnessed spectacular displays of aggressive behavior between males which we describe here.

**Figure 2:**
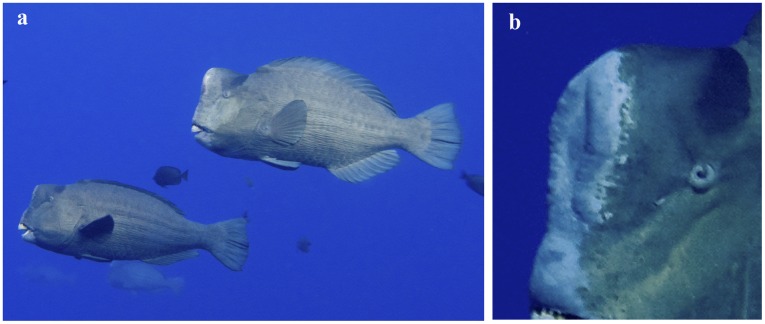
Sexual dimorphism in *Bolbometopon muricatum*. (**a**) Female *Bolbometopon* (lower fish) and male (upper), illustrating dimorphic forehead profile which slopes caudal to beak in females but is nearly parallel with beak in males. Males are also typically larger than females [8]. All observations of courtship and spawning that we observed were between dimorphic fish, suggesting that sex can be determined in the field based on a combination of morphology and behavior [37], [38]. This assumes that most female fish interacting with morphological and behavioral males are indeed female (but see [8,37,39, Muñoz et al. in prep]). (**b**) Detail of male forehead showing ossified ridge characteristic of large males. The ossified ridge and cephalic hump are reduced in females.

**Figure 3:**
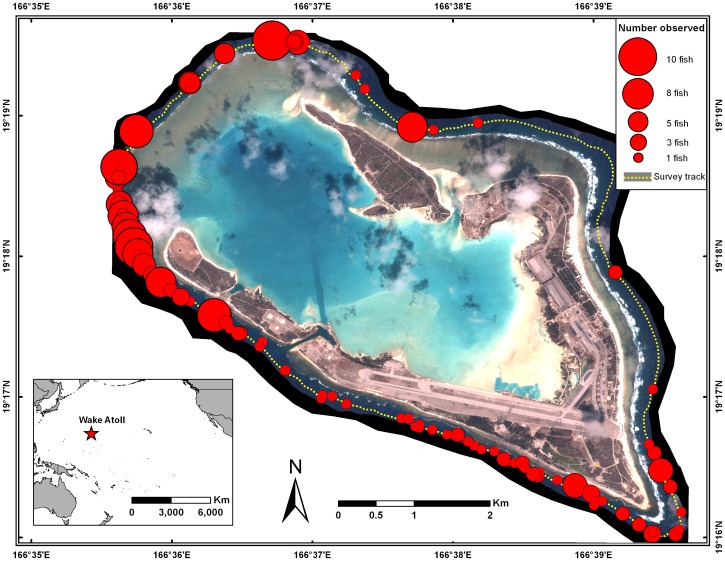
Distribution of *Bolbometopon muricatum* at Wake Atoll observed during towed-diver surveys from 2005–2009. Surveys were conducted on a biennial basis by the NOAA Coral Reef Ecosystem Division. Circles indicate the total number of fish observed at each location around the atoll.

## Results and Discussion

While observing large aggregations of *Bolbometopon* in ∼7 m of water, we heard loud jarring sounds and confirmed they arose from violent impacts between males engaged in repeated, ritualized headbutting behavior (Fig. 1, Video S1, Video S2). During headbutting bouts, males utilized their caudal fins to rapidly collide with their cephalic humps, immediately followed by fast swimming in a semicircle where each fish tried to bite the back and flank of its opponent (Fig. 1d). Following circling, fish swam apart in opposite directions and then turned again face to face to initiate additional collisions. Impact sounds and headbutting were documented on multiple occasions (five and two separate days, respectively) from approximately 0630–0815 h, coincided (in all but one case) with days where we also observed spawning, and were only observed or heard in locations where spawning was also observed. During our study sunrise occurred from 0633–0637 h.

To our knowledge, no scientific documentation of ritualized headbutting exists for marine fishes. *Bolbometopon* is the world’s largest parrotfish and is among the largest of coral reef fishes. How could this dramatic aspect of its social and reproductive behavior have gone unnoticed? We propose two reasons: 1) Low population densities resulting from overfishing dampen competition for resources (females or spawning territories) and/or disrupt the social system [17] so that headbutting contests are uncommon and no longer advantageous. 2) Headbutting contests are common, but negative responses to humans in exploited populations preclude observations of natural behavior. Quantitative estimates of historical abundance are not readily available for *Bolbometopon*, but numerous sources employing indigenous ecological knowledge indicate that precipitous declines in giant bumphead parrotfish populations and decreases in catches correspond with increases in fishing pressure via the advent of spear guns and underwater flashlights [11], [13], [18], [19]. Reports include catching “250 in one night of spearing in shallow water, could catch the whole school on scuba in the 1970’s, and after 1975, 30–50 fish per trip, and nowadays very few.” Another indirect mechanism used to estimate historical numbers is to compare *Bolbometopon* densities relative to areas of low human population density or exploitation levels [11], [12], [20]. With this approach, unexploited areas appear to support 4–48 fish per ha compared with no individuals observed in areas of heavy exploitation.

We hypothesize that geographic isolation and the lack of fisheries exploitation allow historical population densities and traditional spawning sites of *Bolbometopon* to persist. At sites where Bolbometopon are abundant, intense competition [21], sexual selection, and aggressive headbutting contests can be observed. In addition, the protected status of *Bolbometopon* at Wake Atoll results in neutral responses to divers, allowing the unexploited behavioral ecology of this threatened species to be studied.

The context of headbutting in *Bolbometopon* appears similar to the well-known male-male aggressive contests in cetartiodactyls (even-toed ungulates and cetaceans), examples of intrasexual selection where males establish dominance hierarchies or defend territories before mating [22]. As in cetartiodactyls and aggressive contests in general, the physical act of headbutting is likely on the extreme end of a continuum of aggression, with most contests being settled with non-contact displays. For example, male red deer initially roar to settle contests, then proceed to parallel walks, and only later move to violent headbutting/interlocking antlers that carry potential serious costs [23]. We witnessed far more male-male parallel swim displays than we heard impact sounds.

Though rumored to use their forehead to ram corals prior to ingestion [15], the enlarged cephalic hump of *Bolbometopon* may be a classic example of a secondary sexual characteristic resulting from sexual selection (Fig. 2a), such as the massive horns in male bighorn sheep (*Ovis canadensis*). In addition, *Bolbometopon* males exhibit what appears to be an “ossified ridge” on the forehead (Fig. 2b) that may serve a similar function as the cranial appendages of artiodactyls. Any correlations between male hump, body size, and mating success remain to be determined. Freshwater cyprinid (minnows) and mormyrid (elephantfishes) species are reported to headbutt, but males do not display morphological characters (cranial appendages) associated with headbutting (but see breeding tubercles) [24], [25], [26]. In addition, physical contact in these fishes is not confined to the forehead but may also be directed at the body or tail.

Density-dependent alternative mating systems are well known in labroid fishes, but many alternatives only appear at elevated population densities [27] likely resembling conditions in which the systems evolved. Since historical densities have become exceedingly rare for large-bodied species, some alternatives may seldom be observed (a single observation of headbutting by *Bolbometopon* was reported in a recreational dive blog from the Red Sea, an area where *Bolbometopon* is reported to be locally abundant, [11], [28]), and odd morphologies will remain difficult to interpret. Our observations of aggressive headbutting by *Bolbometopon* at Wake Atoll underscore that remote locations and marine reserves, by inhibiting negative responses to human observers and by allowing the persistence of historical conditions, can provide vital opportunities to study ecosystems in their natural state, thereby facilitating the discovery, conservation, and interpretation of a range of behavioral phenotypes.

## Materials and Methods

Permission to conduct field research at Wake Island was granted by Euretha T. Dotson, Major, United States Air Force, Commander, DET 1, 611 ASG, PO Box 68, Wake Island, HI, 96898. All research was conducted in accordance with the Animal Welfare Act (AWA) and with the U.S. Government Principles for the Utilization and Care of Vertebrate Animals Used in Testing, Research, and Training (USGP) OSTP CFR May 20, 1985, Vol.50, No. 97. The study was conducted on free-living wild animals in their natural habitat and solely involved observations of animals and noninvasive measurements.

Wake Atoll (19^o^ 18′ N, 166^o^ 37′ E), is a U. S. Pacific Remote Island, National Wildlife Refuge, and recently designated Marine National Monument co-managed by the U. S. Department of Defense (DOD) and the U. S. Fish and Wildlife Service (USFWS). We conducted 100 h of snorkel and scuba observations of giant bumphead parrotfish (*Bolbometopon muricatum*, Labridae, Scarinae, [29]) from 12–25 August 2011. Visibility ranged from 4.5 m to >30 m, depending on the tidal state (ebbing tide drained the atoll lagoon, decreasing visibility). General underwater conditions can be found in Lobel and Lobel [30]. We chose study sites along the outer fore reef based on the densities of *Bolbometopon* from previous towed-diver surveys by the U. S. National Marine Fisheries Service Coral Reef Ecosystem Division, which conducts biennial surveys of the coral reef ecosystem at Wake Atoll (Fig. 3). Detailed survey methods can be found in Richards et al. [31]. Briefly, divers maneuvered towboards 1–3 m above the substrate and tallied all fishes ≥50 cm TL that entered a 10 m wide swath centered on and forward of the diver. Surveys were 50 min in duration and observational data were recorded in 10 5-min segments. A total of 51 towed-diver surveys were completed during research cruises in 2005, 2007, and 2009. Surveys circumnavigated the island and over 29.64 ha of reef area were surveyed around Wake Atoll during each survey year. The spatial consistency of increased *Bolbometopon* densities in the SW side of the island across years suggests that Bolbometopon may form true fish spawning aggregations at Wake Atoll (*sensu* Domeier [32]); we will present additional analyses that further examine this possibility in a related paper (Muñoz et al. in prep). Because of its remote location and its administration by DOD (since 1934) and USFWS, commercial fishing at Wake Atoll has been excluded and all fishing for *Bolbometopon* is prohibited, so populations can be considered pristine (island-wide mean of 2.97 individuals per ha [SE 0.96], Fig. 1a) [33], [34].

We recorded spawning behavior of *Bolbometopon* using high definition video (Canon Vixia HF S200, Sony HDR-HC9), and still photography (Nikon D300, Canon G9). Observations took place during daylight hours using snorkel, scuba, and towed-diver surveys and were geographically logged with a hand-held GPS [35], [36].

## Supporting Information

Video S1 **Ritualized headbutting of** ***Bolbometopon muricatum*** **at Wake Atoll.** We captured an entire headbutting bout on high definition video, consisting of four, successive charges between two males. The first three resulted in impact (∼5.8/60 s [audible but outside field of view], 19.4/60 s, 26.58/60 s), and the fourth charge resulted in the subordinate male fleeing the contest. Full sequence at normal speed. Given the distinctive sounds from headbutting, once identified, spawning grounds could be monitored with Ecological Acoustic Recorders [40] to assess reproductive effort and aid in the management of this threatened species.
(MP4)

Video S2 **Ritualized headbutting of** ***Bolbometopon muricatum*** **at Wake Atoll.** We captured an entire headbutting bout on high definition video, consisting of four, successive charges between two males. This video shows detail of charges presented in Video S1 at half speed.
(MP4)
